# Hospital Needs

**Published:** 1912-08-24

**Authors:** 


### Hospital Needs.

Specimens of Irish whisky manufactured by Dunville and Co., Limited, Belfast, are well known to hospital authorities, and all who remember our investigations on the subject of alcoholic liquors will need no reminding of the fact that under certain circumstances they are of real dietetic value. The decline in the use of alcohol in hospitals has been partly a reaction against excessive past claims for its use; but the point to look to is the quality and purity of the spirit. Dunville's, whether the "V.R." or "Special Liqueur," will be appreciated by all who enjoy the flavour of thoroughly matured spirit.

